# Binge Alcohol Drinking Alters Synaptic Processing of Executive and Emotional Information in Core Nucleus Accumbens Medium Spiny Neurons

**DOI:** 10.3389/fncel.2021.742207

**Published:** 2021-11-16

**Authors:** Jenya Kolpakova, Vincent van der Vinne, Pablo Giménez-Gómez, Timmy Le, In-Jee You, Rubing Zhao-Shea, Cristina Velazquez-Marrero, Andrew R. Tapper, Gilles E. Martin

**Affiliations:** ^1^Department of Neurobiology, The Brudnick Neuropsychiatric Research Institute, University of Massachusetts Chan Medical School, Worcester, MA, United States; ^2^Department of Biology, Williams College, Williamstown, MA, United States; ^3^Institute of Neurobiology, University of Puerto Rico Medical Sciences Campus, San Juan, PR, United States

**Keywords:** nucleus accumbens, decision making, optogenetics, binge alcohol drinking, synaptic integration, prefrontal cortex, basolateral amygdala

## Abstract

The nucleus accumbens (NAc) is a forebrain region mediating the positive-reinforcing properties of drugs of abuse, including alcohol. It receives glutamatergic projections from multiple forebrain and limbic regions such as the prefrontal cortex (PFCx) and basolateral amygdala (BLA), respectively. However, it is unknown how NAc medium spiny neurons (MSNs) integrate PFCx and BLA inputs, and how this integration is affected by alcohol exposure. Because progress has been hampered by the inability to independently stimulate different pathways, we implemented a dual wavelength optogenetic approach to selectively and independently stimulate PFCx and BLA NAc inputs within the same brain slice. This approach functionally demonstrates that PFCx and BLA inputs synapse onto the same MSNs where they reciprocally inhibit each other pre-synaptically in a strict time-dependent manner. In alcohol-naïve mice, this temporal gating of BLA-inputs by PFCx afferents is stronger than the reverse, revealing that MSNs prioritize high-order executive processes information from the PFCx. Importantly, binge alcohol drinking alters this reciprocal inhibition by unilaterally strengthening BLA inhibition of PFCx inputs. In line with this observation, we demonstrate that *in vivo* optogenetic stimulation of the BLA, but not PFCx, blocks binge alcohol drinking escalation in mice. Overall, our results identify NAc MSNs as a key integrator of executive and emotional information and show that this integration is dysregulated during binge alcohol drinking.

## Introduction

Binge drinking is a potentially dangerous pattern of ingesting large quantities of alcohol over a short period of time that effects primarily late adolescents and young adults with roughly 40% of college students in the United States reporting binge drinking (Krieger et al., 2018). The nucleus accumbens (NAc) is a forebrain region that has long been regarded as a key mediator of the effects of all drugs of abuse, including alcohol (D’Souza, 2015). It receives robust glutamatergic inputs from multiple limbic regions [e.g., basolateral amygdala (BLA) and ventral hippocampus] and from the medial and lateral prefrontal cortex (PFCx) (Groenewegen et al., 1999; Ikemoto, 2007). While the PFCx is responsible for planning, evaluating long-term consequences and is instrumental in retrieving drug-associated memories (Dalley et al., 2004; Zhang et al., 2019), the BLA encodes emotions that shape impulsive behavior and the response to associative learning (Gallagher and Chiba, 1996; Cardinal et al., 2002; Lalumiere, 2014). Importantly, PFCx and BLA send converging synaptic inputs onto single GABAergic medium-spiny neurons (MSNs) (O’Donnell and Grace, 1995; Finch, 1996; French and Totterdell, 2002), the only output cells of the NAc that represent up to 95% of the NAc neuronal population (Meredith and Totterdell, 1999).

Although it has been hypothesized that drug addiction develops as a consequence of imbalances in processing of executive (cortical) and emotional (amygdala) information in the NAc (Bechara, 2005), there are currently no data at the cellular level supporting this theory. Efforts to test this hypothesis at the cellular level have been hampered by the inability to evoke synaptic responses independently from different pathways converging on NAc MSNs using traditional approaches. To circumvent this limitation, we implemented a double-optogenetics approach developed by Klapoetke et al. (2014) using injections of viral-mediated gene expression of Channelrhodopsin-2 and the red-shifted ChrimsonR opsin to evoke excitatory post-synaptic potentials and currents (EPSPs/EPSCs) independently from PFCx and BLA pathways in the same brain slice. We found that NAc MSNs favor the transmission of information from the PFCx in alcohol-naïve mice. Importantly, in binge alcohol drinking mice, NAc MSNs appear to favor BLA information. Also, when tested in freely behaving mice, optogenetic activation of the BLA, but not PFCx, prevented the escalation of alcohol consumption typically observed in mouse models of binge drinking. These data suggest that alteration of the processing of PFCx-driven executive and BLA-driven emotional information by MSNs may be a key cellular mechanism underpinning the role of the NAc in controlling binge alcohol drinking.

## Materials and Methods

### Animals

All experiments were performed using male C57Bl/6J mice. All mice were handled according to the American Association for the Accreditation of Laboratory Animal Care guideline. The protocol was approved by the Institutional Animal Care and Use Committee of University of Massachusetts Medical School. Mice were maintained at constant temperature (22 ± 1°C) and humidity with a 12 h:12 h light–dark cycle. Water and food were provided *ad libitum*.

### Surgeries

We bilaterally (0.6 μl/side) injected 26- to 28-day-old (15–20 g) C57Bl/6J mice with pAAV2-EF1a-hChR2(H134R)-eYFP virus (a gift from Karl Deisseroth, Stanford University, CA) in the PFCx, using a Hamilton syringe with a 2″ long 26 g needle stereotaxically placed into the prelimbic PFCx (+2.0 mm anteroposterior, ±0.35 mm mediolateral (ML), −2.6 mm ventral (DV) from Bregma; Figure 2B). In the same animals, we also bilaterally injected 0.8 μl AAV2-Syn-ChrimsonR-tdTomato (a gift from Edward Boyden; MIT, MA) into the BLA (AP −0.7 mm, ML ±3.3 mm, DV −5.5 mm from Bregma; Figure 2C). The injectors were left in place for 5 min following injection and subsequently raised slowly during an additional 5–8 min. We returned mice to their home cages for 28 days before performing electrophysiological experiments. In a subset of mice (*n* = 6), we injected pAAV2-EF1a-hChR2(H134R)-eYFP (3 mice) and AAV2-Syn-ChrimsonR-tdTomato (3 mice) directly into the NAc (AP +1.5, ML ±1.5, DV −4.3 mm from Bregma) to test the cross stimulation between the two opsins. For both opsins (i.e., ChR2 and ChrimsonR), we chose the AAV serotype 2 to circumscribe infection to the injection sites (Aschauer et al., 2013) and to minimize viral spread (Figures 2B,C).

### Drinking in the Dark Paradigm

Two days following brain viral injection, individually housed mice were allowed to adapt to a reversed light-dark cycle (12 h cycle, OFF at 7AM, ON at 7PM) for one week. Mice were given water bottles with sipper tubes before the experiment to allow habituation and reduce the novelty effect once the ethanol bottle, containing a similar sipper tube, was presented. The total habituation time for the reverse light-dark cycle was 2 weeks before the experiment began. Ethanol exposure started 2 h into the dark phase and lasted for 2 h (Rhodes et al., 2005; Hendrickson et al., 2009). At the start of the experiment, each water bottle was removed and replaced with a pre-weighed 50-mL conical tube containing 20% ethanol with a rubber stopper and double-ball bearing sipper tube. Mice were allowed to drink for 2 h, and then the ethanol bottles were removed, weighed and the water bottles were returned. Ethanol consumed was measured as grams ethanol divided by mouse body weight in kilograms. This protocol was repeated 5 days a week with 2 days off (water only) after each 5-day span. Drip controls were used to account for evaporation and dripping, and experimental bottle weights were corrected using these control values. On average, mice steadily increased their consumption of alcohol before reaching a maximum around 7 g/Kg of 20% alcohol per drinking session at the end of a 2-week period. All electrophysiological recordings were performed 24 h after the last drinking bout during the 4th or 5th post-surgery weeks.

### Slice Preparation

We prepared coronal slices from fresh brain tissue of 8–9 weeks old mice. Following intracardiac perfusion with an ice-cold N-methyl-D-glucamine-based solution (see below), we rapidly removed and transferred the brain in a cold (∼ +0.5°C) oxygenated (95% O_2_ and 5% CO_2_) cutting solution of the following composition (in mM): 95 N-methyl-D-glucamine (NMDG), 2 thiourea, 5 Na^+^-ascorbate, 3 Na^+^-pyruvate to cut slices (300 μm) with a Vibroslicer (VT1200, Leica MicroInstrutments; Germany). Slices were immediately transferred in an incubation chamber and left to recuperate in the NMDG-based solution for 22 min at 32°C before being moved into a chamber containing an artificial cerebrospinal fluid (ACSF; in mM): 126 NaCl, 2.5 KCl, NaH_2_PO_4_.H_2_O, 1 MgCl_2_, 2 CaCl_2_, 26 NaHCO_3_, 10 D-Glucose, at room temperature. Slices were left in this chamber for at least one hour before being placed in a recording chamber and perfused with ACSF at a constant rate of 2–3 ml/min at room temperature (∼21°C). We visualized neurons in infrared differential interference contrast (60×, IR-DIC) videomicroscopy using a fully motorized upright microscope (Scientifica; England).

### Electrophysiology

**FIGURE 1:**
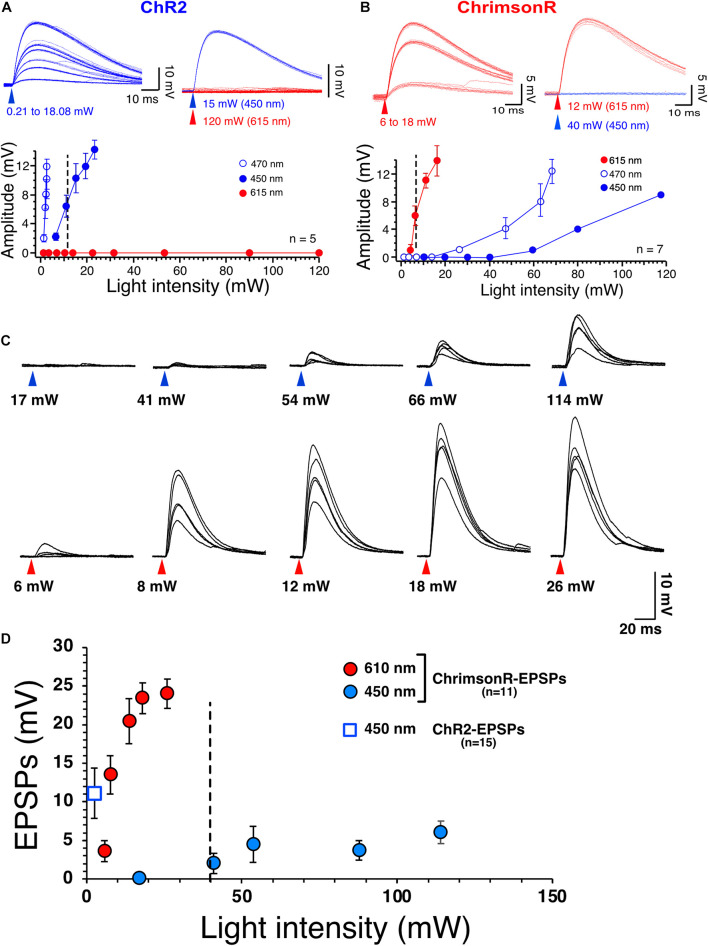
Selective stimulation of ChR2 and ChrimsonR. **(A)** Voltage responses of ChR2 expressed in NAc MSNs to increasing intensities of 450 nm blue light (blue traces) and 615 nm red light (red traces, top right panel). Bottom panel shows graph of ChR2-mediated voltage responses as a function of light intensity at 450 nm (solid blue circles, *n* = 5), 470 nm (open blue circles, *n* = 5) and 615 nm light intensity (solid red circles, *n* = 5). Note the lack of sensitivity of ChR2 to red light. **(B)** Voltage responses of ChrimsonR expressed in NAc MSNs to increasing intensities of 615 nm (red traces, *n* = 7) and 450 nm blue light (blue traces, top right panel, *n* = 7). Bottom panel shows graph of ChrimsonR-mediated voltage responses as a function of light intensity at 450 nm (solid blue circles, *n* = 7), 470 nm (open blue circles) and 615 nm light intensity (solid red circles). Average values in graphs are expressed as mean ± standard error (SEM). **(C)** Representative EPSPs following injection of AAV-ChrimsonR in PFCx in response to increase intensity to 450 nm (blue triangles) and 610 nm (red triangles) light. Note that blue light evokes EPSPs at intensity higher than 40 mW. **(D)** Graph showing ChrimsonR-mediated EPSP amplitude as a function of increasing light (450 – solid blue squares and 610 nm – solid red squares) intensities. Open blue square shows the average amplitude of ChR2-mediated EPSPs as a function of low 450 nm light intensity (0.78 mW).

Slices were prepared according to method previously described (Ji et al., 2015). Whole-cell patch clamp recordings were performed in the absence of GABA receptor antagonists (Ji et al., 2017). Briefly, borosilicate glass electrodes (1.5 mm OD, 5–7 MΩ resistance) were filled with an internal solution containing (mM): 120 K-methanesulfonate; 20 KCl; 10 HEPES; 2 ATP, 1 GTP, and 12 phosphocreatine. Following seal rupture, series resistance was 18.3 ± 1.1 MΩ in a randomly selected sample of 23 MSNs, fully compensated in current clamp recording mode, and periodically monitored throughout recording sessions. Recordings with changes of series resistance larger than 20% were rejected, as were MSNs with a resting membrane potential more positive than −80 mV. Voltage and current traces in whole-cell patch-clamp were acquired with an EPC10 amplifier (HEKA Elektronik; Germany). Sampling was performed at 10 kHz and digitally filtered voltage and current traces were acquired with PatchMaster 2.15 (HEKA Elektronik; Germany) at 2 kHz. All traces were subsequently analyzed off-line with FitMaster 2.15 (HEKA Electronik; Germany). EPSP/Cs were evoked optically by flashing 1 ms-long 450 nm blue and 610 nm red lights through the light path of a microscope 60× objective using independent high-powered LEDs (pE-100 470 and 610 nm CooLED, NY, United States) under the control of the acquisition software (PatchMaster, HEKA, Germany). To rapidly switch (i.e., milliseconds) between blue and red lights, single bandpass filters (450/50 and 620/50 from Chroma, Bellows Falls, VT) were placed directly in front of the optical path of each light source mounted on a two-way exchanger (CooLED, NY, United States) at the back of the microscope. Light intensity was measured at slice level. Although gating strength was not a function of the amplitude of the first EPSPs (see Figure 3), we adjusted the light intensity to evoke synaptic events of at least 5 mV in amplitude. However, if more than 35 mW was necessary to evoke ChR2-driven EPSPs, recordings were rejected based on the sensitivity of ChrimsonR to blue light (Figure 1B). Ethanol was obtained from Sigma-Aldrich (Saint Louis, MO, United States).

**FIGURE 2:**
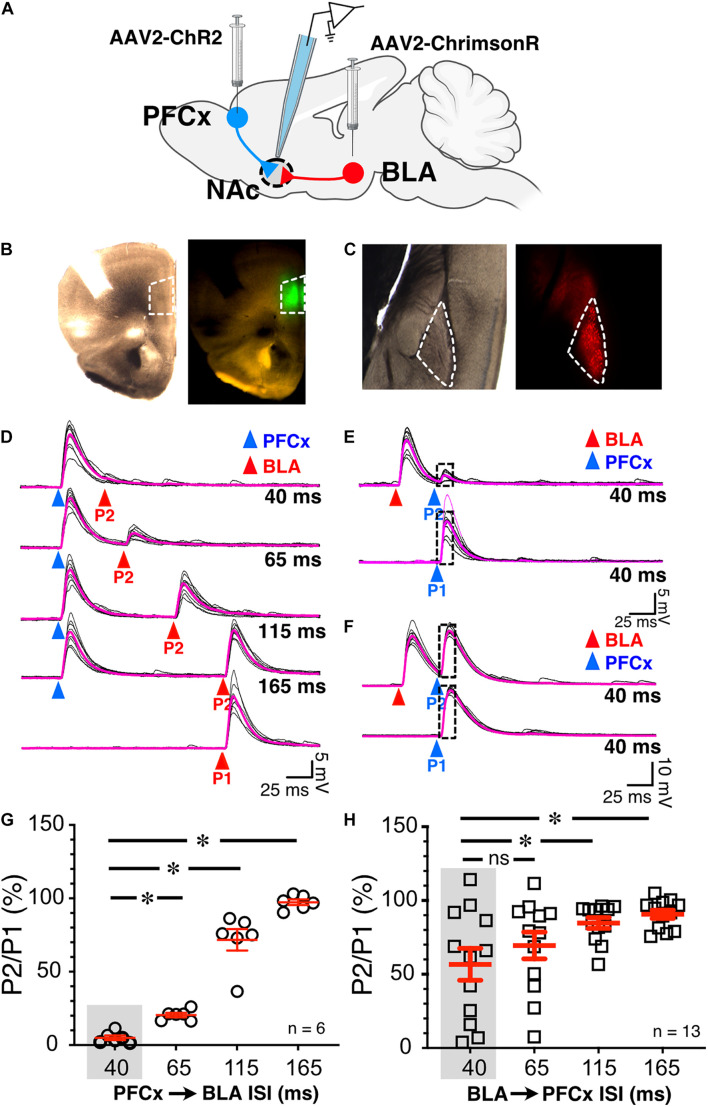
Synaptic gating between BLA and PFCx inputs is bidirectional and asymmetric in alcohol-naïve mice in NAc MSNs. **(A)** Cartoon illustrating the injections and the recording sites. **(B)** Bright field (left panel) and epifluorescence of AAV2-ChR2-eYFP injected in the prelimbic prefrontal cortex area outlined by white broken line. **(C)** Bright field (left panel) and epifluorescence of AAV2-ChrimsonR-tdTomato injected in the amygdala area outlined by white broken line. Note that the infection is confined to the targeted areas. **(D)** 10 overlaid traces of PFCx-EPSPs (blue arrowheads) evoked before stimulation of the BLA pathway (red arrowheads, P2) at increasing interstimulus intervals (from 40 to 165 ms, ISI). As a control, the lowest traces (red P1) show BLA-EPSPs evoked in the absence of PFCx-EPSPs. **(E,F)** 10 overlaid traces of BLA- EPSPs (red arrowheads) evoked before PFCx-EPSPs (blue arrowheads, P2) at a 40 ms interval. While BLA-EPSPs inhibit PFCx-EPSPs in some MSNs **(B)** at this short interval, it fails to do so in others **(C)**. **(G)** Circles show the magnitude of the gating of BLA-EPSPs (P2) by PFCx inputs (PFCx → BLA) at various intervals expressed as percent relative to control (P1) in 6 MSNs. **(H)** Square symbols show magnitude of the gating of PFCx-EPSPs (P2) by BLA inputs (BLA → PFCx) at various intervals relative to control (P1) in 13 MSNs. Each symbol presents a MSN. Average values in graphs are expressed as mean ± standard error (SEM). ^∗^ *p* < 0.01. Purple traces show averaged EPSPs in panels **(D–F)**.

**FIGURE 3:**
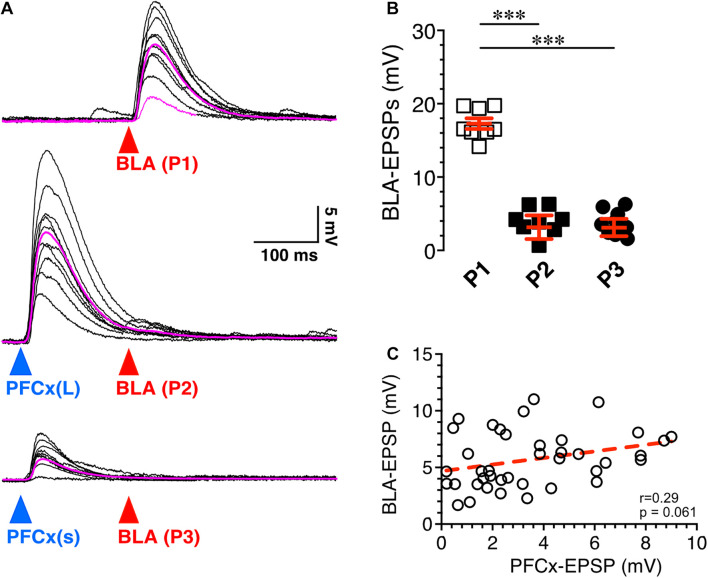
Synaptic gating is a sensitive phenomenon. **(A)** Ten overlaid BLA EPSPs (red arrowhead) evoked in absence (top traces) and in presence of large (middle traces) and small (bottom traces) PFCx-EPSPs (blue arrowheads) in the same MSN (*n* = 8). Purple traces show averaged EPSPs. **(B)** Amplitude of BLA-EPSPs in absence (open squares) and in presence of large (solid black squares, P2) and small (solid circles, P3) PFCx-EPSPs. **(C)** Relationship between amplitude of BLA-EPSPs as a function of PFCx-EPSPs amplitudes. Average values in graphs are expressed as mean ± standard error (SEM). ^∗∗∗^ *p* < 0.001.

### Optogenetic Stimulation in Freely Moving Mice

28–32 days old mice were injected with 0.6 μl of pAAV5-CaMKII-hChR2(H134R)-eGFP (UNC viral core) bilaterally either in the prelimbic PFCx or BLA and on the same day implanted with a fiber optic fiber (Doric Lenses, CA) located above the NAc (AP +1.5, ML ±1.5, DV −4.0 mm from Bregma). We checked injection sites and fiber optics placement at the end of experiments. Mice were allowed to recover for 9 days before being transferred into the testing chamber. Three days after transfer, mice were connected to the fiber optic cables. For habituation purposes, mice remained tethered to the fiber optic cables and LED drivers. Following 4 days of habituation, mice were exposed to alcohol according to the Drinking in the Dark (DID) protocol as described above. Alcohol drinking behavior was quantified by measuring licks of the drinking spout. Drinking behavior (number and timing of licks) was measured in mice exposed to 2 h of the DID paradigm. Following 4 days of drinking without stimulation, the PFCx or BLA was optogenetically stimulated (ChR2, 5 mW 5 ms-long 470 nm light pulses at 10 Hz with alternating 2-min stimulation ON and 2-min stimulation OFF) throughout the 2 h drinking interval. The comparison was made between the last day of alcohol drinking without stimulation (Day 4) and the last day of alcohol drinking with stimulation (Day 8). We chose 10 Hz based on the capacity of glutamatergic terminals to follow this frequency (Figure 7B; 10 Hz). In contrast, 20 Hz stimulation evoked failures to transmit glutamate (data not shown). For saccharine control experiment, 0.3% saccharine solution was made and consumption measured during the same optogenetic stimulation protocol.

### Analysis

**FIGURE 4:**
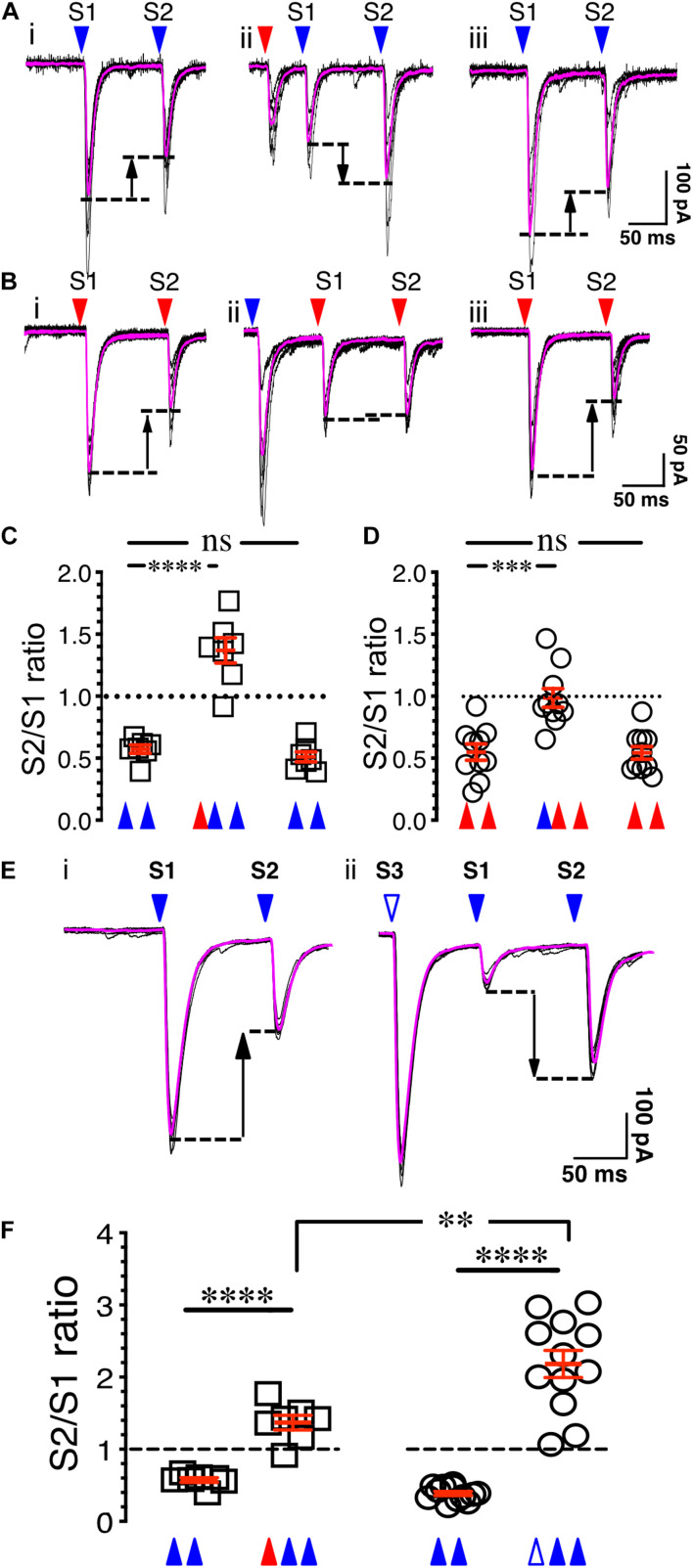
Synaptic gating is a pre-synaptic phenomenon. **(Ai)** Eight overlaid traces of PFCx-EPSCs (blue arrowheads) evoked 100 ms apart (S1 and S2). **(Aii)** Same as in i with stimulation of the BLA afferents (red arrowhead) preceding PFCx-EPSCs. **(Aiii)** Shows in the same recording that paired pulse depression returned to control level when stimulation of BLA afferents was turned off. **(B)** Stimulation order was reversed from panel **(A)**. **(C)** Graph showing the switch from depression to facilitation following stimulation of BLA inputs (*n* = 7). **(D)** Graph showing the loss of depression following stimulation of PFCx inputs (*n* = 10). Note that the magnitude of the switch is not similar. **(Ei)** Eight overlaid traces of PFCx-EPSCs (blue arrowheads) evoked 100 ms apart (S1 and S2). **(Eii)** PFCx inputs were stimulated 70 ms before the PPR protocol (S3). Purple lines show average traces. **(F)** Graphs showing the switch from depression to facilitation when the PPR at cortical synapses is preceded by stimulation of the BLA (circles) or PFCx (squares) pathways (*n* = 11). Average values in graphs are expressed as mean ± standard error (SEM). ^∗∗^ *p* < 0.01, ^∗∗∗^ *p* < 0.001, ^****^ *p* < 0.0001. Purple traces show averaged EPSCs in panels **(A,B,E)**.

**FIGURE 5:**
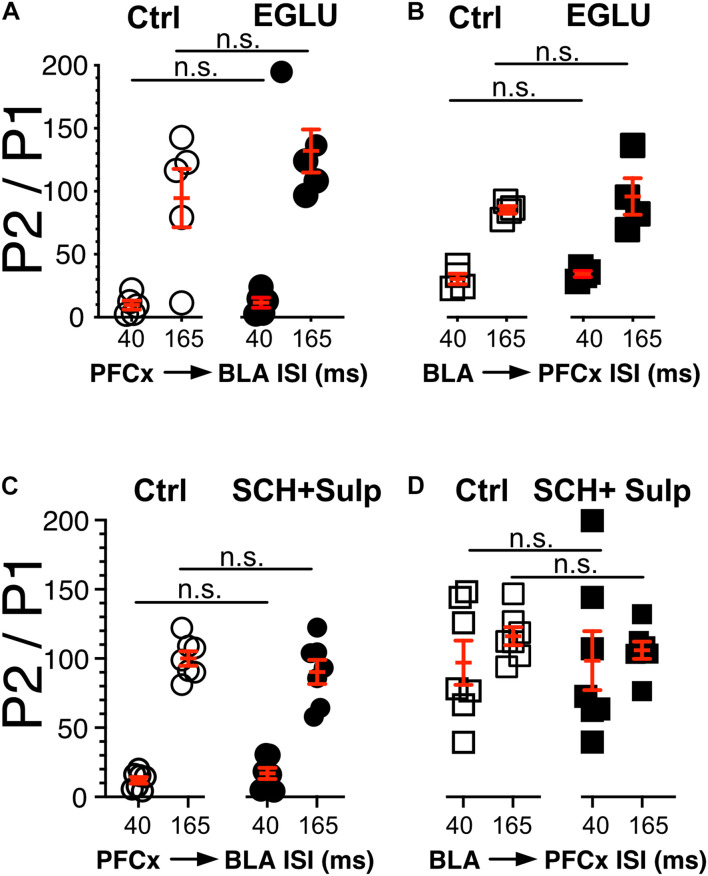
Neither DA nor metabotropic receptors control synaptic gating. **(A,B)** Circles show the magnitude of the gating of BLA-EPSPs (P2) by PFCx inputs (PFCx → BLA) at various intervals expressed as percent relative to control (P1) in 5 MSNs before and during exposure to EGLU. Square symbols show the magnitude of the gating of PFCx-EPSPs (P2) by BLA inputs (BLA → PFCx) at various intervals expressed as percent relative to control (P1) in 5 MSNs before and during exposure to EGLU. **(C,D)** Same as panels **(A,B)** in presence of SCH-23390 and Sulpiride.

**FIGURE 6:**
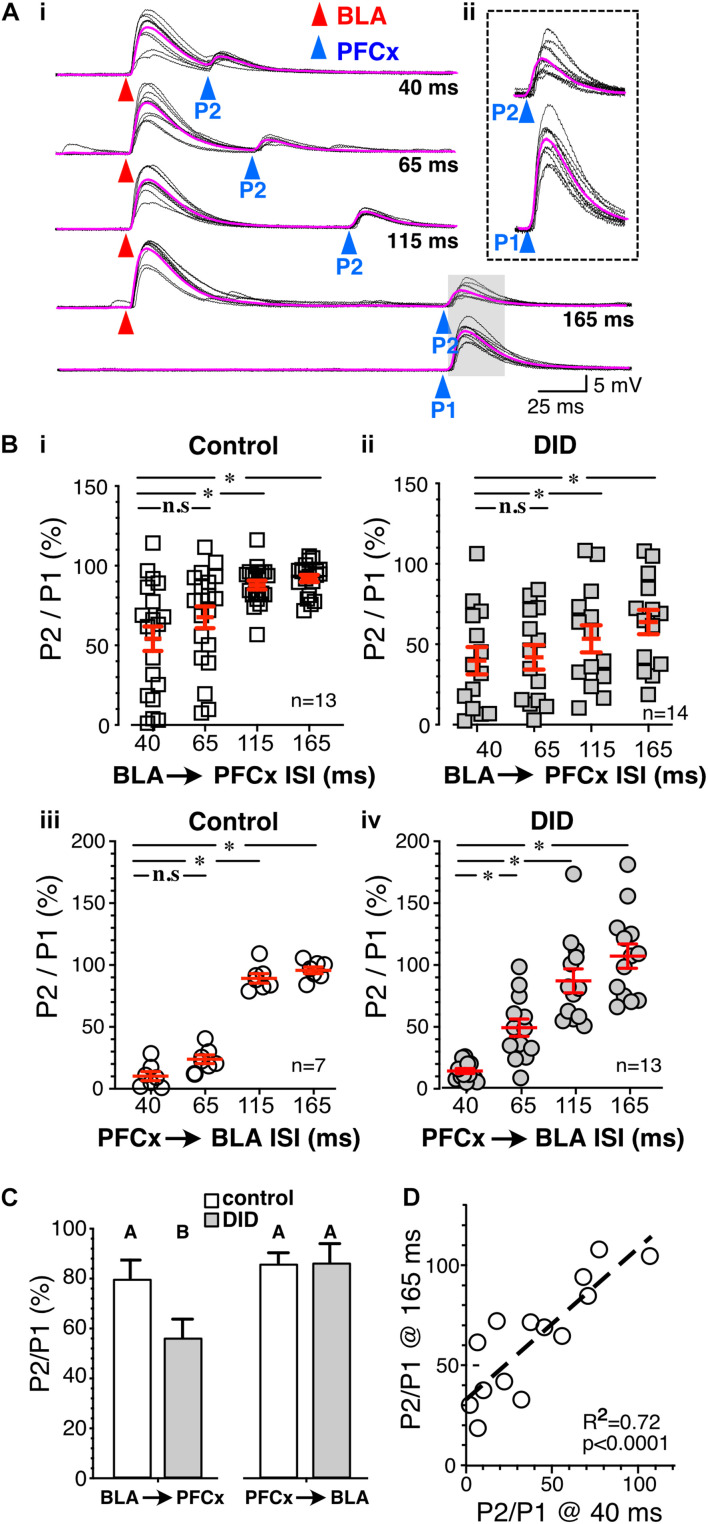
Binge drinking strengthens BLA-driven inhibition of PFCx inputs. **(Ai)** Ten overlaid traces of PFCx-evoked EPSPs (blue arrowheads) in absence (P1-bottom panel) or presence of BLA-EPSPs (red arrowheads) in the same MSN from an EtOH drinking mouse. Purple traces show average EPSPs. **(Aii)** Enlarged PFCx-EPSPs traces (P2) evoked 165 ms after BLA-EPSPs or in its absence (P1) as shown in shaded area in panel **(Ai)**. Note the persistent gating at 165 ms interval indicated by the vertical arrow. **(Bi,ii)** Magnitude of gating of BLA → PFCx inputs in water-drinking (Control) and Drinking-in-the-Dark mice (DID) at various intervals relative to control expressed as percent relative to control (P1) in 13 and 14 MSNs, respectively. **(Biii,iv)** Magnitude of gating of PFCx → BLA inputs in water-drinking mice (Control) and Drinking-in-the-Dark mice (DID) at various intervals relative to control in 7 and 13 MSNs, respectively.**(C)** Summary statistics of Panel **(B)**. Change in EPSP amplitude (P2/P1) resulting from BLA → PFCx and PFCx → BLA gating is altered by binge alcohol drinking (DID). Averages and SEMs represent GLM model estimates of the effects of gating direction and alcohol exposure. Groups identified with different letters are significantly different. **(D)** Correlation between the magnitude of inhibition of PFCx-EPSPs by BLA at 40 ms and that measured at 165 ms interval in DID mice. Average values in graphs are expressed as mean ± standard error (SEM). ^∗^ *P* < 0.01.

**FIGURE 7:**
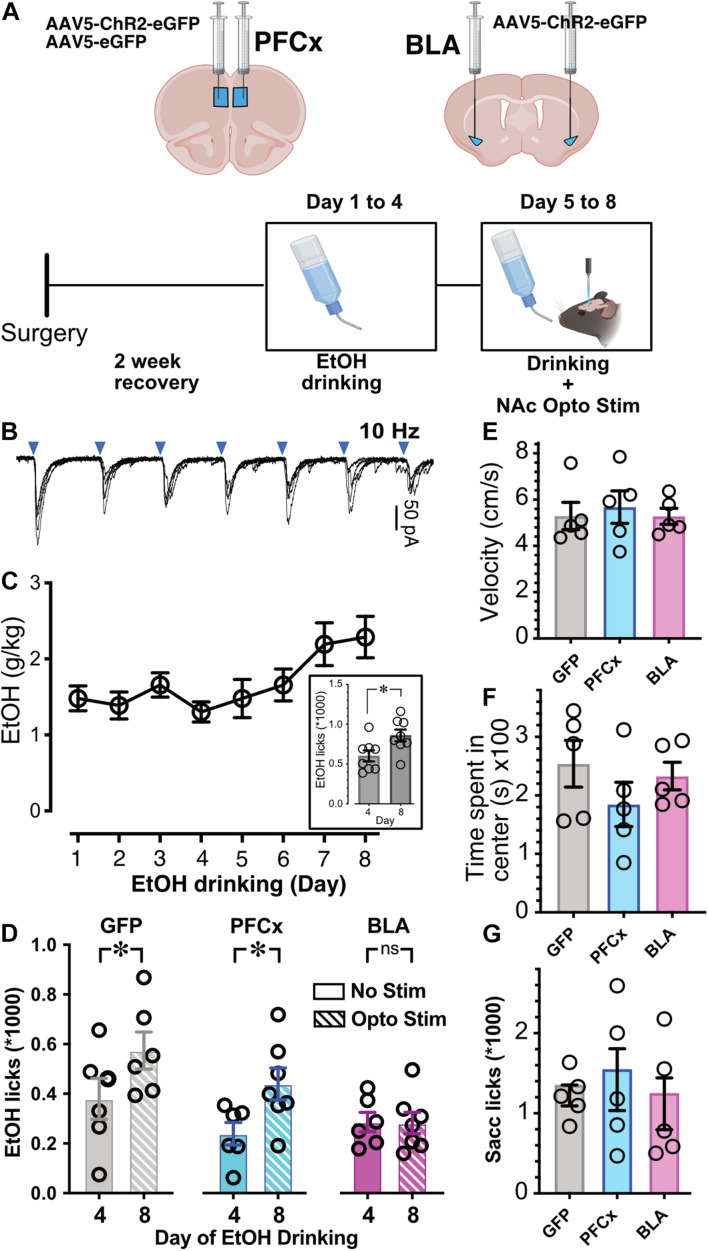
Effects of optogenetic activation of PFCx or BLA inputs on EtOH drinking. **(A)** Schematic illustrating the sites of bilateral injections and the timeline of the behavioral experiments. **(B)** Synaptic responses to 10 Hz light stimulation of PFCx afferents. Blue triangles indicate time of stimulations. **(C)** EtOH consumption over a period of 8 consecutive days. Note the escalation between the 4th and last day. Graph in inset shows the corresponding number of licks at Day 4 and 8. **(D)** Number of licks measured over a 2 h period at Day 4 and 8 in mice injected with GFP in the PFCx (GFP, gray columns), with ChR2 in the PFCx (PFCx, blue columns), or in the BLA (BLA, pink columns) in absence (solid columns, no stim) or presence of light stimulation (hatched columns, Opto Stim). **(E)** Velocity measured in mice injected with GFP (GFP) or ChR2 (BLA; PFCx). **(F)** Time spent in center of field in mice injected with GFP (GFP) or ChR2 (BLA; PFCx). Average values in graphs are expressed as mean ± standard error (SEM). Each symbol represents a mouse. **(G)** Number of licks of 0.3% saccharine in 2 h in mice injected with GFP (GFP) or ChR2 (BLA; PFCx). ^∗^ *P* < 0.01.

EPSP/Cs maximum amplitude was measured in a 20 ms time window 10 ms after the onset of the stimulus. In each experimental condition, 10 consecutive EPSP/Cs evoked every 15 s were measured. Data are expressed as mean ± SEM. Electrophysiological and behavioral data were analyzed with Prism 7.0 (Graphpad, United States) Statistics package using either *t* tests or one- and two-way ANOVAs (Figures 3–5) or with mixed-effects general linear model (Figures 2, 6, 7; SAS JMP 7.0) to account for random effect variables such as cell ID, direction of stimulation, and alcohol treatment, as indicated in the text. The drinking behavior of freely moving mice was quantified by summing the number of drinking spout licks per 2-min interval with intermittent optogenetic stimulation LED light ON or OFF. Statistical comparisons assessed the total number of licks over the 2 h drinking interval per day. The criterion for statistical significance was *p* < 0.05 for all experiments.

## Results

### Selective Responses of Accumbens Core Medium Spiny Neurons to Stimulation of ChR2 and ChrimsonR

The present study necessitated independent stimulation of ChR2 and ChrimsonR in the same brain slices with minimal stimulation overlap. In view of this requirement, the selectivity of blue light to evoke ChR2- and red light to evoke ChrimsonR-mediated responses was optimized in brain slices of mice injected in the NAc with one of the viral plasmids. Accumbens core MSNs from injected mice had a mean resting membrane potential and membrane input resistance of −86.7 ± 0.6 mV and of 117.47 ± 14 MOhm, respectively, indicative of healthy neurons (*n* = 23 randomly selected neurons). As expected, low intensity (i.e., <5 mW) 470 nm blue light readily evoked rapid (∼300–400 μs delay) non-synaptic depolarizations of MSNs in slices of mice injected with pAAV2-EF1a-hChR2(H134R)-eYFP. Depolarization increased in amplitude with increasing light intensities (Figure 1A, graph, open circles). Importantly, 610 nm red light failed to elicit responses, even at the highest intensity (i.e., 120 mW) tested (Figure 1A top panel, red traces), demonstrating strong selectivity of ChR2 for blue light (Figure 1A, graph, solid red circles). A different group of mice were injected with AAV2-Syn-ChrimsonR-tdTomato into the NAc. Short (1 ms) red light pulses evoked depolarizations with a rapid onset like that of ChR2-mediated responses (Figure 1B). However, 470 nm blue light also evoked ChrimsonR-mediated depolarization when the intensity was higher than 30 mW (Figure 1B, graph, open circles), showing cross-stimulation between blue and red light for ChrimsonR. To decrease the sensitivity of ChrimsonR to blue light, the blue light was left-shifted by replacing the blue light filter (470/20 nm) with a single bandpass filter (450/50 nm). Although this new filter did not totally render ChrimsonR insensitive to the left-shifted blue light, it markedly decreased its sensitivity. Thus, only blue light with an intensity greater than 40 mW and higher excited ChrimsonR (Figure 1B, solid blue circles). We then tested ChrimsonR-mediated excitatory post-synaptic potentials’ (EPSPs) sensitivity to 450 and 610 nm at PFCx’s synapses. We found that their sensitivity is very similar to that of non-synaptic ChrimsonR (Figure 1B), with blue light evoking small amplitude EPSPs at light intensity higher than 40 nm (Figure 1C, top traces and D solid blue circles). In contrast, red light evoked large synaptic responses (Figure 1C, bottom traces and D, solid red circles). Also, we compared these synaptic responses to ChR2-mediated EPSPs at the same synapse. We found that 450 nm stimulation evoked robust EPSPs at very low intensity (Figure 1D, open blue square). In a random sample of 12 MSNs, we measured ChR2- and ChrimsonR-EPSC kinetics and found no statistical differences between the two groups, with ChR2-EPSCs decay being 7.72 ± 0.42 ms while that of ChrimsonR was 7.58 ± 0.37 ms. This result suggests that the populations of AMPA receptors activated by cortical and amygdala inputs are likely similar in terms of their subunit composition.

### Synaptic Gating Between Cortical and Amygdala Inputs in the Core Nucleus Accumbens of Alcohol-Naïve Mice

To test whether cortical inputs gate BLA-mediated EPSPs, we bilaterally injected AAV2-ChR2-eYFP and AAV2-ChrimsonR-tdTomato into the PFCx and BLA areas, respectively, and recorded from NAc MSNs receiving *both* PFCx and BLA inputs (Figure 2A). The spread of the virus was well contained to the injection sites (Figures 2B,C). We employed a protocol where we evoked PFCx-EPSPs (Figure 2D, blue triangles) followed by BLA-EPSPs (PFCx → BLA gating; Figure 2D, P2 - red triangles) at different time intervals (i.e., 40, 65, 115, and 165 ms). For each interval, we averaged 10 consecutives traces recorded with 15 s between trials. As a control, 10 BLA-EPSPs were evoked in the absence of PFCx-EPSPs (Figure 2D, P1 - red triangle, bottom traces) with changes in BLA-EPSP amplitude represented as the ratio of P2/P1 × 100%. At a short (40 ms) interval, BLA-EPSPs were almost completely inhibited by preceding stimulation of the PFCx pathway (Figure 2D top traces and 2G, 4.9 ± 1.5% of control). As intervals lengthened, the amplitude of BLA-EPSPs increased, with amplitudes returning to control levels at a 165 ms interval (Figure 2D lower traces and Figure 2G, 97.3 ± 1.9% of control). This pattern was consistently and repeatedly observed in each of the 6 MSNs recorded. As the length of the interval between the PFCx and BLA stimulation increased, the inhibitory effects of PFCx inputs on BLA-induced EPSPs became significantly weaker [Figure 2G; *F*(3,15) = 132.7, *p* < 0.0001, general linear model (GLM), with cell ID as random variable]. Tukey *post hoc* test showing 40 ms interval being significantly different from all other intervals. This result demonstrates that PFCx afferents exert a powerful but narrow time-dependent inhibition of BLA projections synapsing on core NAc MSNs.

Using the same protocol, the stimulation order was subsequently reversed with BLA-EPSPs preceding PFCx-EPSPs (BLA → PFCx gating). PFCx-EPSPs evoked 40 ms after BLA-EPSPs were similarly strongly inhibited in some MSNs (Figure 2E) although this effect was not observed in all cells. Indeed, in some MSNs, the gating was either weaker or simply lacking at the same 40 ms interval (Figure 2F). When averaged over 13 MSNs, the mean inhibition of BLA → PFCx gating was 56.7 ± 10.81% of control at a 40 ms interval, and 90.6 ± 2.9% at 165 ms. There was also significant difference between the intervals tested [Figure 2H; *F*(3,36) = 10.4, *p* < 0.0001, GLM with cell ID as a random variable], with Tukey *post hoc* test revealing significant difference between 40 and 115 ms intervals and 40–165 ms intervals.

Interestingly, the BLA → PFCx gating response was significantly different from that observed in PFCx → BLA gating (Figures 2G vs. H). When comparing the interaction between the interval and the stimulation order, we found a significant interaction effect [*F*(3,54.9) = 8.9, *p* < 0.0001, GLM with cell ID as a random variable], with Tukey *post hoc* tests revealing BLA → PFCx significantly weaker inhibition than PFCx → BLA gating at 40 and 65 ms intervals.

Next, we tested whether the magnitude of PFCx → BLA gating depended on the size of PFCx-EPSPs. Stimulation of BLA inputs alone evoked large EPSPs (Figure 3A top traces), that were robustly inhibited by large PFCx-EPSPs (Figure 3A middle traces). Interestingly, lowering the intensity of blue light reduced the amplitude of PFCx-EPSPs without altering gating efficacy (Figure 3A bottom traces), a pattern observed in all 8 recorded MSNs (Figure 3B). Furthermore, we found no correlation between the amplitude of PFCx- and BLA-EPSPs with a slope was not significantly different from zero (40 ms delay; Figure 3C). Overall, this shows that the magnitude of gating, at least at BLA synapses, does not appear to be a function of the strength of PFCx synaptic transmission.

### Synaptic Gating Between Cortical and Amygdala Inputs Is a Pre-synaptic Phenomenon

To determine whether the control of glutamate synaptic transmission of the PFCx pathway by BLA inputs is pre-synaptic, the probability of release of PFCx terminals was examined. Since paired-pulse ratio (PPR) is a protocol commonly used to assess the probability of release (Dobrunz and Stevens, 1997), we hypothesized that if gating is pre-synaptic, stimulation of one pathway should alter the PPR of the other. To that end, the probability of release of cortical afferents was assessed by evoking PFCx-EPSCs 100 ms apart (Figure 4A). For each MSN, 8 consecutive current traces were recorded with a delay of 15 s between traces. Confirming previous findings (Ji et al., 2015), stimulation of the PFCx pathway with an interstimulus interval of 100 ms led to a paired pulse depression (PPD) with the second EPSCs being smaller than the first (Figure 4Ai), indicative of an initial high probability of release (Dobrunz and Stevens, 1997). When averaged over 7 MSNs, the PPR of S2/S1 was 0.57 ± 0.03 (Figure 4C). Interestingly, when the PPR at PFCx synapses was preceded by stimulation of BLA inputs (red arrowhead, Figure 4Aii) with an delay of 65 ms, a value we chose based on Figure 2H showing that this interval induces a roughly 50% reduction of PFCx-EPSPs by BLA inputs, the paired-pulse depression significantly switched to paired-pulse facilitation (PPF, Figure 4Aii), with a S2/S1 ratio of 1.37 ± 0.10 [Figure 4C, *n* = 7; *F*(2,12) = 75.6, *p* < 0.0001, RM one-way ANOVA], an effect that was reversed when stimulation of the BLA inputs was turned off (Figures 4Aiii,C, *p* = 0.71, Tukey test). This result is in line with the hypothesis that BLA inputs synapse pre-synaptically on PFCx afferents and decrease the probability of release of glutamate.

Whether PFCx inputs similarly affected PPR at BLA synapses was tested subsequently. Stimulating BLA afferents 100 ms apart led to a robust depression with a S2/S1 ratio of 0.54 ± 0.03 (Figures 4Bi,D, *n* = 10). Interestingly, preceding PFCx-EPSCs, with a delay of 80 ms a value based on data of Figure 2G, essentially blocked the depression at BLA synapses [Figure 4D; *F*(2,18) = 19.4, *p* < 0.0001, RM one-way ANOVA], but this time without inducing facilitation (Figures 4Bii,D), with a S2/S1 ratio of 0.98 ± 0.07 (Figure 4D). As with the control of PFCx PPR by BLA inputs, this effect quickly dissipated when stimulation of PFCx inputs was turned off (Figures 4Biii,D). This demonstrates that pre-synaptic control of BLA glutamate release by PFCx inputs was significantly weaker than the control of PFCx glutamate release by BLA afferents (PPR = 0.98 ± 0.7 for BLA vs. PPR = 1.37 ± 0.10 for PFCx; *t* = 3.1, *df* = 15, *p* = 0.0072, Student’s *t* test).

To assess whether the changes in synaptic gating underlying the pre-synaptic control of glutamate release from PFCx terminals by the BLA are similar to the changes in PPR, stimulation of BLA inputs (Figure 4Aii) was replaced with stimulation of PFCx afferents. Although three consecutive blue light pulses led to a similar reversal of the PPR from depression to facilitation (Figures 4Ei vs. ii, F, *t* = 9.5, *df* = 11.5, *p* < 0.0001, Welch-corrected *t* test), the magnitude of the effect was markedly larger compared to what was observed following BLA pathway stimulation (Figure 4F). Thus, while BLA stimulation induced a paired-pulse facilitation with a S2/S1 ratio of 1.37 ± 0.10 (*n* = 7, Figure 4F, red triangle), this effect was significantly larger (*t* = 3.8, *df* = 15.9, *p* = 0.0016, Welch-corrected *t* test) when BLA stimulation was replaced with stimulation of PFCx afferents (Figure 4F, S2/S1 ratio of 2.31 ± 0.24, white triangle). These observations show that gating appears to induce an increase in PPR that indicates decreased probability of release. It also suggests that a partial, but not full overlap in mechanisms or it might simply be attributable to the fact that direct depolarization of the PFC input is likely more efficacious at engaging the exact same mechanisms that are engaged by BLA stimulation.

### Pre-synaptic Dopamine and Metabotropic Glutamate Receptors Do Not Control Synaptic Gating in Nucleus Accumbens Medium Spiny Neurons

In light of the findings showing that gating is pre-synaptic, we investigated its nature by testing the putative involvement of dopamine and type 2/3 metabotropic glutamate receptors, two families of receptor known to control glutamate release (Pennartz et al., 1992; Manzoni et al., 1997; Martin et al., 1997; Nicola and Malenka, 1997; Wang et al., 2012). We measured the strength of gating at 40 and 165 ms intervals before (Ctrl) and during exposure to 100 μM EGLU, a selective mGluR2/3 antagonist when stimulation of PFCx pathway preceded that of BLA inputs (PFCx → BLA, Figure 5A), and the reverse (BLA → PFCx, Figure 5B). We observed no statistically significant change in gating strength in either condition. In a different group of mice, we tested the effects of dopamine D1 and D2 receptor antagonist 5 μM SCH 23390 and 1 μM Sulpiride. Like with EGLU, the combination of both antagonists failed to significantly alter synaptic gating in both direction (i.e., PFCx → BLA; Figure 5C and BLA → PFCx; Figure 5D). These results indicate that gating is not mediated by dopamine or mGluRs. Nor it is mediated by NMDA receptors as the antagonist d-APV (10 μM) also failed to modify gating (data not shown).

### Binge Alcohol Drinking Alters Synaptic Gating in an Input Specific Manner

Having established the basic principles and mechanisms underlying synaptic gating of PFCx and BLA inputs to NAc MSNs, gating sensitivity was subsequently assessed in mice exposed to repeated binge alcohol drinking. Following a 2-week period of binge alcohol drinking, and 24 h after the last drinking bout, the reciprocal control of glutamate synaptic transmission in NAc MSNs was examined at PFCx and BLA synapses. First, BLA → PFCx gating was measured at the same intervals of 40, 65, 115, and 165 ms. Interestingly, BLA → PFCx gating was substantially altered by alcohol exposure. Strikingly, at 165 ms interval many DID cells never reached 100% P2/P1 ratio (Figures 6Ai,ii), a feature that distinguished it from all other cells measured. At the shortest interval of 40 ms, the inhibition in DID mice was variable, ranging from 2.3 to 106.3%, with an average of 56.7 ± 10.9% (Figure 6Bii, DID), mirroring that of alcohol-naïve mice that ranged from 1.13 to 121.8% of control with an average of 39.8 ± 8.5% (Figure 6Bi, Control). However, at longer intervals (i.e., 115 and 165 ms), inhibition of PFCx-EPSPs by BLA afferents in DID mice remained highly variable with some MSNs showing weak inhibition of PFCx-EPSPs (Figures 6Ai,ii,Bii); a result that contrasted sharply with synaptic gating at the same interval in alcohol-naïve mice where only a consistently weak inhibition was observed (Figure 6Bi). Thus, in some MSNs from DID mice, the inhibition persisted at the longest interval of 165 ms, while it was nearly absent in other MSNs (Figure 6Bii). At 165 ms interval recorded from alcohol-exposed mice, the inhibition ranged from 18.7 to 107.9% (Figure 6Bii), with an average value of 63.8 ± 7.6% of control, showing a stronger inhibition than that of alcohol-naive mice (93.6 ± 2.4% of control, compare Figure 6Bi Control and Figure 6Bii DID). Accompanying the stronger inhibition, we observed a significantly larger variance at intervals 110 and 165 ms. Additionally, in DID BLA → PFCx gating, a significant positive correlation was detected between the magnitude of the EPSP inhibition at 40 ms and 165 ms [Figure 6D; *F*(1,12) = 30.69; *p* < 0.0001], indicating a faithful gating mechanism throughout different interval progression.

Next, PFCx → BLA gating was measured. As in water-drinking mice (Figure 6Biii, Control), PFCx inputs robustly and consistently inhibited BLA-EPSPs to 10.6 ± 2.0% of control (*n* = 7) at the shortest interval tested in DID mice (40 ms. Figure 6Biv); a value that was similar to control mice (10.5 ± 3.7% of control, Figure 6Biii). Inhibition got weaker with longer intervals and was absent at 165 ms (103.4 ± 9.7% of control; Figure 6Biv). PFCx → BLA gating was thus mostly unaffected by binge alcohol drinking. However, when analyzing the variance at each interval between Control and DID mice, we found a significant effect of binge drinking on the variability PFCx gating of BLA inputs at all but 40 ms intervals between Control and DID mice (Table 1). While binge alcohol drinking does not on average alter the gating of BLA inputs by PFCx, it does increase the variability at 65, 115 and 165 ms intervals, suggesting an effect, albeit smaller than the one observed for gating of PFCx inputs by BLA.

**TABLE 1:** *F*-test of Two-Sample Variance.

		Mean		Variance			
			
		DID	Control	DID	Control	*F* value	*p*-value
*BLAx* → *PFC*	*40 ms*	39.8	56.7	1012.5	1568.9	0.65	0.2220
*BLAx* → *PFC*	*65 ms*	41.9	65.0	808.9	843.9	0.96	0.4676
*BLAx* → *PFC*	*115 ms*	53.4	88.4	992.9	141.2	7.03	0.0009
*BLAx* → *PFC*	*165 ms*	63.8	93.6	801.3	74.3	10.78	0.0001
*PFCx* → *BLA*	*40 ms*	10.6	10.6	52.9	95.9	0.55	0.1793
*PFCx* → *BLA*	*65 ms*	45.6	24.2	645.3	83.6	7.72	0.0100
*PFCx* → *BLA*	*115 ms*	83.4	89.5	1208.6	101.4	11.92	0.0031
*PFCx* → *BLA*	*165 ms*	103.5	96.1	1244.5	52.7	23.63	0.0005

To compare all the groups in Figure 6B and to determine whether alcohol treatment alters the weight between PFCx and BLA inputs, we tested for the interaction between the order of stimulation and the presence of alcohol treatment. We indeed observed a significant interaction between the order of stimulation (PFCx or BLA first) and alcohol treatment, [Figures 6B,C; *F*(1,162.2) = 10.9, *p* = 0.0012, MM GLM]. Tukey *post hoc* revealed significant differences between control and EtOH BLA → PFCx groups, but no differences between Control and EtOH PFCx → BLA groups. Interestingly, we did not observe the effect of alcohol on the intervals (*p* = 0.56), suggesting that the relationship between intervals is not perturbed by the alcohol treatment.

In summary, we have found that 2-week EtOH DID treatment significantly affected weight contribution between PFCx and BLA inputs by altering the overall strength of BLA → PFCx gating but not that of PFCx → BLA gating, while additionally significantly affecting the variability in both pathways.

### Suppression of Binge Alcohol Drinking by Optogenetic Basolateral Amygdala Activation in Freely Moving Mice

Next, we wanted to determine whether activation of PFCx or BLA pathways to NAc has behavioral significance. Although we could not recapitulate the *in vitro* double stimulation “gating” protocol, we wanted to know whether single pathway stimulation would be sufficient to alter mouse binge alcohol consumption. We injected mice with pAAV5-CaMKII-hChR2(H134R)-eGFP either into the PFCx or BLA nuclei, with controls injected with a GFP expressing AAV into the PFCx and fiber optic probes placed in the NAc core (Figure 7A). Optogenetic stimulation of PFCx and BLA pathways was achieved *via* delivery of 10 Hz 470 nm light, 2 min on, 2 min off (Figure 7B). As described previously (Ji et al., 2017), alcohol access over a 2-week period produces an escalation in consumption. When we repeated this experiment over eight consecutive days, we also saw a significant increase between day 4 and day 8 of alcohol consumption in a group of *n* = 8 mice that did not undergo surgery (Figure 7C; *t* = 2.5, *df* = 14, *p* = 0.0235). Since we have observed this increase in consumption, we exposed mice to 20% EtOH for 4 days, and then optogenetically stimulated during EtOH drinking days 5–8 (Figure 7A). Binge alcohol drinking behavior was quantified by recording the number and timing of licks of the drinking spout on day 4 of EtOH consumption (last day before stimulation) and day 8 (last day of stimulation). Like mice that did not undergo surgery (Figure 7C, inset), mice injected with AAV-GFP showed an increase of EtOH consumption on day 8 after stimulation (Opto Stim, patterned columns) compared to day 4 without stimulation (No Stim; Figure 7D, GFP). Injection of AAV-ChR2 into the PFCx showed a pattern like GFP controls: an escalation of drinking on stimulation day 8 (Opto Stim, patterned columns) compared to day 4 without stimulation (No Stim; Figure 7D, PFCx). Interestingly, in mice injected with AAV-ChR2 into the BLA using the same paradigm, optogenetic stimulation prevented this escalation in EtOH consumption on day 8 (Opto Stim; Figure 7D BLA). We found a significant interaction between light presence and the brain area injected [Figure 7D; *F*(2,17.6) = 3.6, *p* = 0.0497, with Day and Mouse ID as random variables, GLM]. *Post hoc* Student’s *t* test showed significant differences in PFCx and GFP groups between day 4 (no stimulation day) and day 8 (stimulation day), with no differences in BLA-injected mice. This finding was in line with our patch clamp data showing an increased efficiency of the gating of PFCx-EPSPs by BLA activation. Also, on Day 4 there is no differences between GFP, PFCx, and BLA group. Finally, comparison of alcohol consumption at DAY 8 between the GFP, PFCx, and BLA groups showed a highly significant difference between GFP and BLA but not between GFP and PFCx groups. This effect was not due to a general depression of behavioral activity in BLA-stimulated mice, as no differences in movement velocity [Figure 7E; *F*(1,12) = 0.4885, *p* = 0.6252] and time spent in the center [Figure 7F; *F*(2,12) = 0.4103, *p* = 0.6724] were observed in an open-field test. Importantly, 0.3% saccharine drinking behavior was unaffected by PFC- or BLA-stimulation [Figure 7G; *F*(2,12) = 1.256, *p* = 0.3198], thus demonstrating the BLA-specific inhibition of alcohol drinking as an alcohol-specific effect.

## Discussion

The NAc is considered to be a key mediator of the effects of drugs of abuse such as alcohol (Scofield et al., 2016). Within the NAc, MSNs serve as integrators of potentially conflicting drives (Lara and Wallis, 2015; Wassum and Izquierdo, 2015; Klenowski, 2018) and receive inputs from different brain regions such as the PFCx and BLA while projecting to the effector cells (Humphries and Prescott, 2010). The present study confirms this function of NAc MSNs as integrators of different inputs with the PFCx and BLA synapsing onto the same core NAc MSNs. Importantly, the relative timing of input signals determines the subsequent MSNs’ output. BLA and PFCx inputs reciprocally inhibit each other, but the strength of this reciprocal inhibition is asymmetric, with PFCx → BLA gating stronger than the reverse. The interactions underlying PFCx → BLA and BLA → PFCx gating occur pre-synaptically. Surprisingly, preceding exposure to binge alcohol drinking uni-directionally strengthens BLA → PFCx gating in a selective manner (Figure 6). Overall, these findings suggest the integration of different inputs in the MSNs as a potential target for modifying substance abuse behaviors. In line with this suggestion, optogenetic activation of BLA inputs in the NAc does indeed result in an inhibition of the increase in alcohol drinking behavior observed during the first days of alcohol exposure in mice.

The idea that afferents originating in the cortex, amygdala, hippocampus and thalamus converge onto the same NAc MSNs is supported by multiple anatomical (French and Totterdell, 2002, 2003) and electrophysiological recordings in anesthetized rats (O’Donnell and Grace, 1995; Finch, 1996; Brady et al., 2005; Calhoon and O’Donnell, 2013), and in acute tissue slices (Stuber et al., 2011). However, the degree of convergence remains unclear with some studies showing convergence of these inputs in less than 5% of MSNs (Callaway et al., 1991; Finch, 1996) while others reported a strong convergence in nearly all MSNs (O’Donnell and Grace, 1995), a discrepancy that may be attributed to the use of different recording methods (i.e., intra vs. extracellular recordings). Our data supports strong convergence since we found that the vast majority (i.e., ∼81%) of core NAc MSNs received inputs from both the PFCx and the BLA.

The current work illustrates that a key feature of synaptic gating between PFCx and BLA afferents is its bidirectionality, with inhibition of synaptic transmission of one pathway by the other being mirrored when the stimulation order is reversed. This phenomenon is reminiscent of recent findings by Calhoon and O’Donnell showing a similar bidirectional gating between thalamic and PFCx afferents in anesthetized rats (Calhoon and O’Donnell, 2013). Furthermore, the delay of 50 ms at which inhibition was the strongest in this study was similar to the one reported in our study (i.e., 40 ms), suggesting that synaptic gating of PFCx-, BLA-, and thalamic-inputs converging onto NAc MSNs shares characteristics at least in terms of its timing. One major difference, however, is the need for high frequency stimulation to induce gating in the intact brain while a single EPSP was sufficient to obtain similar strong inhibition in our *in vitro* slice study. This difference might reflect a higher efficiency to evoke synaptic events in slices compared to the intact brain. Surprisingly, our data shows that not only is a single PFCx-EPSP sufficient to block the transmission of BLA information, but that this inhibition can even be induced by very small PFCx-EPSPs, illustrating the high sensitivity of synaptic gating between cortical and amygdala inputs in NAc MSNs. A possible explanation for the apparent discrepancy between slice- and intact-brain recordings is that seemingly small somatic EPSPs are much larger at their point of inception (i.e., the synapse). As EPSPs travel to the soma following synaptic induction distally, they get subjected to the strong filtering by the dendrites’ cable properties, resulting in a depression of the detected EPSPs amplitude (Rall, 1959). Overall, the current findings demonstrate the bidirectional nature of gating different inputs to the NAc and underscores the extreme sensitivity of this process.

Our efforts to identify the pre-synaptic mechanism responsible led to the conclusion that neither DA receptors and mGluRs nor NMDA receptors are recruited during synaptic gating. This is not entirely surprising considering synaptic gating kinetics that is characterized by a very fast onset, with one input blocking the other within a few ms before disappearing after 165 ms. This time course is rather incompatible with slower acting a G-protein coupled receptor/second messenger system. Because this result suggested the participation of pre-synaptic ionotropic receptors, we were surprised to observe the lack of effects of the NMDA receptor antagonist d-APV. Therefore, it is likely that pre-synaptic control is mediated by AMPA receptors. Unfortunately, testing this hypothesis would require neutralizing these receptors, which we cannot without blocking EPSPs altogether.

Bechara (2005) proposed that an underlying cause for drug addiction is the hyperactivity in the amygdala system that overrides the reflective system of the prefrontal cortex. The present study examined how MSNs reconcile drastically different information originating specifically from the medial prelimbic PFCx and the BLA. To determine whether the integration of these conflicting inputs has physiological relevance, binge alcohol drinking was assessed in freely behaving mice with optogenetically activated PFCx- or BLA-inputs to the NAc. Stimulation of BLA projections, but not PFCx inputs, in the NAc abolished the increase in alcohol drinking behavior over the first 4 days of alcohol exposure. Interestingly, using a Pavlovian conditioning protocol, Millan et al. (2017) reported that activation of the BLA to NAc pathway similarly blocked alcohol consumption triggered by cue-conditioned alcohol seeking (Millan et al., 2015). Additionally, in an interesting parallel, our electrophysiological data shows that alcohol exposure that strengthens BLA control of PFC inputs has lesser effect on PFCx gating of BLA. The alteration of BLA input to the NAc responsible for modifying alcohol consumption behavior might be the result of changes in direct synapses with MSNs or the inhibition of PFCx projecting inputs to MSNs. While our *in vivo* experiment could not distinguish between these two modes, our electrophysiological data showed that alcohol exposure disrupts the sensitive balance underlying the integration of PFCx and BLA information by NAc MSNs. Based on these electrophysiological observations, we hypothesize that BLA activation can modify alcohol drinking behavior through the inhibition of PFCx inputs to MSNs in the NAc. It is important to note that the interpretation of our behavioral data is limited by the fact that we could not replicate the gating protocol (i.e., double stimulation) used in *in vitro* slice recordings. In particular, the 10 Hz frequency could alter synaptic transmission in a way unrelated to the gating mechanism unveiled in slices (Pascoli et al., 2014) and may cause a suppression of BLA synaptic efficacy possibly accounting for the confounding effect on binge drinking (i.e., blockade instead of increase of alcohol consumption Figure 7D). Further studies recruiting both pathways with stimulation intervals based on our electrophysiological findings in freely moving mice are needed to fully understand the respective roles of PFCx and BLA pathways in controlling binge alcohol drinking. Another limitation of our behavioral design was that we could not distinguish between the effect of time and stimulation on binge alcohol drinking. Additional studies need to be performed to tease apart their respective influence.

## Conclusion

We propose a model where PFCx and BLA afferents synapse on the same MSNs and inhibit each other reciprocally. PFCx afferents exert a stronger control over glutamate release from BLA inputs than the reverse in alcohol-naïve mice. Upon repeated alcohol exposure, the control of PFCx inputs by BLA afferents is strengthened at longer intervals, a mechanism that may be responsible for lower alcohol consumption following the stimulation of BLA afferents in freely moving mice.

## Data Availability Statement

The raw data supporting the conclusions of this article will be made available by the authors, without undue reservation.

## Ethics Statement

The animal study was reviewed and approved by UMASS Medical School Institution on Animal Care and Use.

## Author Contributions

JK, AT, and GM designed the experiments. JK, I-JY, PG-G, TL, RZ-S, CV-M, and GM performed the experiments. JK, VV, and GM analyzed data. GM wrote the manuscript with input from other authors.

## Conflict of Interest

The authors declare that the research was conducted in the absence of any commercial or financial relationships that could be construed as a potential conflict of interest.

## Publisher’s Note

All claims expressed in this article are solely those of the authors and do not necessarily represent those of their affiliated organizations, or those of the publisher, the editors and the reviewers. Any product that may be evaluated in this article, or claim that may be made by its manufacturer, is not guaranteed or endorsed by the publisher.
